# Developing community-based urine sampling methods to deploy biomarker technology for the assessment of dietary exposure

**DOI:** 10.1017/S136898002000097X

**Published:** 2020-12

**Authors:** Amanda J Lloyd, Thomas Wilson, Naomi D Willis, Laura Lyons, Helen Phillips, Hayley G Janssen, Martina Stiegler, Long Xie, Kathleen Tailliart, Manfred Beckmann, Leo Stevenson, John C Mathers, John Draper

**Affiliations:** 1 Institute of Biological, Environmental and Rural Sciences, Aberystwyth University, Aberystwyth SY23 3DA, UK; 2 Human Nutrition Research Centre, Institute of Cellular Medicine, Newcastle University, Newcastle-upon-Tyne, UK; 3 Faculty of Education, Health & Community, Liverpool John Moores University, Liverpool, UK

**Keywords:** Dietary exposure, Metabolomics, Biomarkers, Home urine collection, Population monitoring, Cost-effective diagnostic tool

## Abstract

**Objective:**

Obtaining objective, dietary exposure information from individuals is challenging because of the complexity of food consumption patterns and the limitations of self-reporting tools (e.g., FFQ and diet diaries). This hinders research efforts to associate intakes of specific foods or eating patterns with population health outcomes.

**Design:**

Dietary exposure can be assessed by the measurement of food-derived chemicals in urine samples. We aimed to develop methodologies for urine collection that minimised impact on the day-to-day activities of participants but also yielded samples that were data-rich in terms of targeted biomarker measurements.

**Setting:**

Urine collection methodologies were developed within home settings.

**Participants:**

Different cohorts of free-living volunteers.

**Results:**

Home collection of urine samples using vacuum transfer technology was deemed highly acceptable by volunteers. Statistical analysis of both metabolome and selected dietary exposure biomarkers in spot urine collected and stored using this method showed that they were compositionally similar to urine collected using a standard method with immediate sample freezing. Even without chemical preservatives, samples can be stored under different temperature regimes without any significant impact on the overall urine composition or concentration of forty-six exemplar dietary exposure biomarkers. Importantly, the samples could be posted directly to analytical facilities, without the need for refrigerated transport and involvement of clinical professionals.

**Conclusions:**

This urine sampling methodology appears to be suitable for routine use and may provide a scalable, cost-effective means to collect urine samples and to assess diet in epidemiological studies.

Nutrition is a major determinant of health throughout the life-course, and eating patterns have a significant impact on the risk of developing common complex diseases, including CVD, type 2 diabetes, dementia and several cancers^(1,2)^. Consequently, healthy eating advice and interventions to improve dietary choices are at the core of many public health information strategies internationally^(3–5)^. The measurement of habitual food intake and the assessment of individual nutritional status provide core information for monitoring population health, and have been used for exploring the relationships between lifestyle choices and health outcomes and in the design of clinical trials^(6)^. However, because of the complexity of eating patterns and the conceptual and practical difficulties in recording or recalling the types and amounts of foods and beverages consumed, errors in self-reporting of dietary intakes by cognitively-able individuals are commonplace and substantial^(7–9)^. Such problems may be exacerbated when individuals consume meals out-of-the-home or eat ready-made meals and other pre-prepared foods because they may not know the individual ingredients in these foods or are unable to estimate portion sizes accurately^(10)^. In addition, the instruments used for self-reporting of diet – for example, FFQ, 24-h recalls, or diet diaries – impose a significant burden both on the individuals reporting their eating behaviour as well as on the researchers subsequently calculating food and nutrient intake. Furthermore, the most vulnerable members of the society who are at greatest risk of malnutrition (very old individuals, young children) encounter most problems with self-reporting, and thus, alternative or complimentary approaches to monitoring diet would have a substantial value^(11–13)^.

To address these issues, there has been considerable recent interest in the discovery and validation of metabolites derived from individual foods present in urine samples (or other biofluids), which provide biomarkers of dietary exposure and whose measurement may mitigate the limitations of traditional dietary assessment methodologies by providing objective estimates of food consumption^(14–16)^. However, to provide robust evidence of dietary exposure, such biomarker technology demands the development of urine sampling methods that ensure high compliance by populations. Urine collection and sampling kits need to be simple for participants to use in their home settings with minimal impact on their day-to-day activities and which would yield samples that allow a comprehensive and reliable quantitation of the targeted biomarkers. Many research studies requiring accurate measurements of exposure biomarkers have adopted the ‘gold standard’ method of requesting participants to collect all urine over a 24-h period^(17)^. However, spot urine samples are much less burdensome to collect than 24-h urine, and there is also a risk that full 24-h collection may not be achieved in all cases, leading to inaccurate and misleading results. Recently we showed that spot urine samples representing specific temporal phases of the day can substitute adequately for 24-h urine samples^(18)^ for biomarker discovery and habitual dietary exposure measurements^(19,20)^.

Most common procedures for community-based urine sampling require either a dedicated visit by participant/patients to a clinical research centre (CRC) to drop off urine samples, a home visit by a research assistant^(21)^, or a courier service to pick up samples^(22)^. As well as incurring significant costs for travel or transport, such approaches impose logistical challenges, may interfere with the normal daily activities of study participants and/or place substantial time demands on CRC staff. Additionally, to avoid deterioration of the chemical composition of urine during transport, cooling or refrigeration has been employed, which adds further cost. Although the use of chemical preservatives to inhibit the growth of contaminating microbes in urine samples is commonplace^(23–27)^, many of these compounds are strongly ionic and may interfere with analytical methods based on MS. In the current article, we report the outcomes of investigations of the feasibility and acceptability of community-based procedures for collection, sampling, preservation and transport of urine samples that are designed to be cost-effective, scalable and suitable for use in large epidemiological studies or for national dietary surveys of populations.

## Methods

### Study design and urine sampling methods

**Fig. 1:**
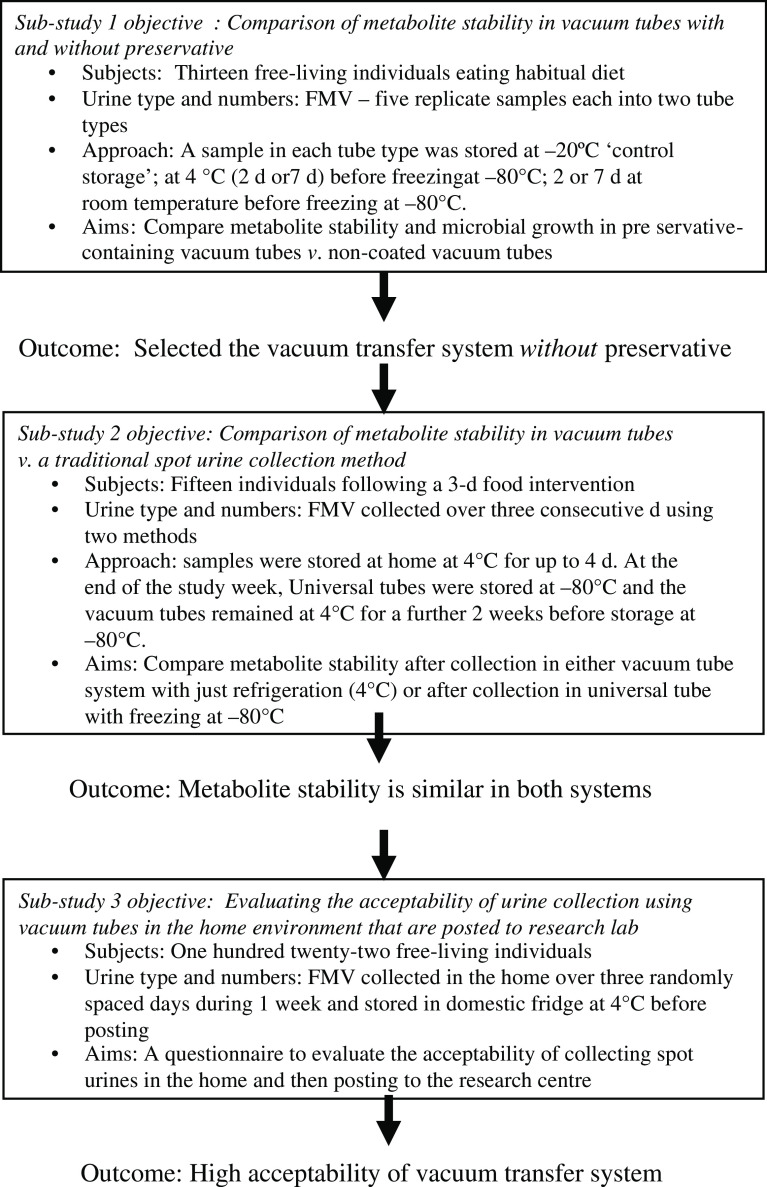
Schematic of the overall study design. FMV, first morning void

The overall study comprised of three independent sub-studies, which aimed to investigate the utility of vacuum transfer tubes for sampling of spot urines for dietary biomarker analysis in a home environment (Fig. 1). *Sub-study 1* and *sub-study 2* were concerned with evaluating the compositional stability of urine samples under different collection and storage conditions. Previous research indicated that an analysis of nine independent urine samples would provide sufficient statistical power for metabolome comparisons^(18,28)^. However, when dealing with people, there is usually more chance of error, drop-outs, non-compliance, etc.; therefore, we aimed to recruit 12–15 individuals for both studies. *Sub-study 3* recruited 122 free-living individuals and used an online questionnaire to assess the acceptability of an optimised spot urine sampling method using the vacuum transfer system in the home environment followed by posting to an analytical laboratory.

- *Sub-study 1: Comparison of metabolite stability in vacuum tubes with and without preservative*

The stability of urine chemistry when collected in a vacuum tube containing a lyophilised preservative (Becton and Dickinson Vacutainer^®^ urinalysis preservative tube; chlorhexidine, ethyl paraben and sodium propionate) was compared with that in a non-coated vacuum tube. In an in-house study in Aberystwyth, thirteen healthy individuals (eight male, five female; one smoker and twelve non-smokers; age 25–60) were recruited and asked to continue consuming their habitual diet. They collected the first morning void (FMV) urine at home and dispensed this into five replicate preservative-containing vacuum tubes and five replicate non-coated vacuum tubes using the vacuum transfer method, utilising a plastic collection vessel (100 ml) along with a separate transfer straw (Becton and Dickinson, as illustrated in online Supplementary Material 1D). These samples were stored at 4°C in the participants’ domestic fridges and then transported to the research facility to be subjected to a series of storage treatments that mimicked conditions likely to be encountered if samples remained for several days in a domestic environment. A sample in each tube type was stored at –20°C, deemed as ‘control storage’ to mimic the conditions typical of a domestic freezer. A sample in each tube type was subjected to the following four storage conditions: at 4°C (2 or 7 d) before freezing at –80°C; 2 or 7 d at room temperature (RT) before freezing at –80°C.

- *Sub-study 2: Comparison of metabolite stability in preservative-free vacuum tubes v. a traditional spot urine collection method*

Fifteen, free-living individuals (eight male, seven female; non-smokers; age 21–74) were recruited from the Human Nutrition Research Centre, Newcastle University database. Each participant collected FMV urine samples at home using the vacuum transfer system (see online Supplementary Material 1C) as well as the traditional plastic jug and universal tube method (see online Supplementary Material 1A). Samples were collected using both methods on three consecutive days during which the participants consumed different meals as part of a separate dietary intervention study (see online Supplementary Material 2), reported elsewhere^(29)^. Written instructions on how to collect FMV urine samples using both methods were provided for the fifteen participants, but no verbal one-to-one guidance was given. All samples were stored at home at 4°C for up to 4 d and then brought to the research facility in Newcastle in a cooler bag at the end of the study week. Universal tubes were stored immediately at –80°C and the vacuum tubes remained at 4°C for a further 2 weeks before storage at –80°C. Samples were then transported to the analytical facility in Aberystwyth on dry ice for metabolite stability analysis.

- *Sub-study 3: Evaluation of the acceptability of vacuum transfer system for urine collection in the home environment*

In a third study, we recruited 122 healthy volunteers (twenty-eight male, ninety-four female; smokers and non-smokers; age 18–64) by text message and e-mail invitation after a large-scale survey on eating habits. These volunteers were free-living and were asked to maintain their habitual diet. A kit containing a urine collection container, transfer straw, vacuum tubes (as shown in online Supplementary Material 1D, where there was enough vacuum tubes to collect three randomly spaced FMV urines over a week) and a Royal Mail Safebox™ were posted to each individual. Urine samples were collected and stored at home at 4°C and then posted back in the Royal Mail Safebox™. The Safeboxes had prepaid first-class postage, with the aim to arrive back at the research centre within 1–2 working days. The volunteers were asked to complete an online questionnaire about the acceptability of aspects of spot urine collection methodology (see online Supplementary Material 3). The online questionnaire had thirteen questions, which asked participants to rank the extent of their agreement with each statement on a five-point scale from ‘strongly disagree’ to ‘strongly agree’. Responses were analysed as a percentage of overall feedback.

### Optical density measurement to assess bacterial growth

After storage treatments, the urine samples were mixed by vortexing, and 100-μl aliquots, in duplicate, were added to ninety-six-well flat-bottomed microtiter plates. The optical density of samples was determined using a Hidex Sense Microplate Reader (model 425-301), with absorbance set at 600 nm. Samples were read three times in Hidex PlateReaderSoftware (version 0.5.11.0) at 37°C, with agitation between readings.

### Urine sample normalisation

All urine samples were normalised by refractive index prior to analysis to ensure all MS measurements were made within a similar dynamic range. Samples were defrosted overnight in a 4°C fridge. Once defrosted, samples were centrifuged (600 ***g*** for 5 min at 4°C), placed on ice, and aliquots of thawed urine (1000 µl) were transferred into labelled 2-ml Eppendorf tubes. An OPTI Hand Held Refractometer (Bellingham Stanley™ Brix 54 Model) was calibrated with deionised water (dH_2_O) and dried with paper tissue according to the manufacturer’s instruction. Following this, 220 µl of sample was transferred onto the refractometer dish; its specific gravity (SG) was recorded in triplicate and temperature was noted. The refractometer was rinsed with dH_2_O between samples and dried with paper tissue.

Based on these figures, aliquots of required amounts of urine were diluted with dH_2_O in 2-ml Eppendorf tubes to make up to a total volume of 500 µl. Extraction was performed by adding 500 µl of pre-chilled MeOH (extraction-grade, Fisher Scientific). Samples were vortexed, then placed on an orbital shaker (FATSM002; Favorgen Biotech Corp.) for 20 min at 1400 rpm and 4°C in the dark. All extracted samples were stored at –80°C until further analysis.

### Non-targeted flow infusion electrospray ionisation high-resolution MS

Urine samples were analysed using flow infusion electrospray ionisation high-resolution MS (FIE-HRMS) to generate a non-targeted metabolome fingerprint. For this purpose, 20 µl of extracted sample was transferred to a glass HPLC vial containing a 200-µl flat-bottom micro insert (Chromacol). All samples were diluted with 80 µl H_2_O:MeOH (3:7) directly in the vial. Mass spectra were acquired on an Exactive Orbitrap (ThermoFinnigan) mass spectrometer coupled to an Accela (ThermoFinnigan) ultra-performance liquid chromatography system. Twenty microlitres of the diluted sample was injected and delivered to the electrospray source via a flow solvent (mobile phase) of premixed HPLC-grade MeOH (Fisher Scientific) and ultra-pure H_2_O (18·2 Ω) at a ratio of 7:3. The flow rate was 200 μl/min ‘for the first 1·5 min and 600 μl/min ‘for the remainder of the analysis. The total run time was 3·0 min.

Positive and negative ionisation modes were acquired simultaneously. For each ionisation mode, one scan event was used to acquire all mass spectra, 55·000–1000·000 and 63·000–1000·000 *m/z* for positive and negative mode, respectively. The scan rate was 1·0 Hz. Mass resolution was 100 000, with automatic gain control 5 × 10^5^ and maximum injection time 250 ms, for both ionisation modes. Following data acquisition, raw profile data (.raw; ThermoFinnigan) was converted to the .mzML open file format and centroided^(30)^ using msconvert (TransProteomicPipeline)^(31)^. All further processing of mzML files was performed using the R Statistical Programming Language^(32)^.

Dimensionality reduction of the acquired mass spectra was performed by taking each *m/z* value from scans about the apex of the infusion profile and binning the *m/z* and intensity values at 0·01 amu intervals. The result was a *n* × *p* matrix, where *n* is the sample and *p* is the *m/z* feature and cells are the respective average intensity values.

### Targeted metabolite quantification using ultra-HPLC and MS

Absolute concentrations of selected dietary exposure biomarkers (see table in online Supplementary Material 4) were measured using ultra-HPLC (UHPLC) triple quadrupole (QQQ) MS operating in multiple reaction monitoring (MRM) mode^(20,29)^. MRM chromatograms were acquired on a TSQ Quantum Ultra QQQ mass spectrometer (ThermoFinnigan) equipped with a heated electrospray ionisation source and coupled to an Accela UHPLC system.

The UHPLC system was equipped with either a Thermo-Scientific Hypersil Gold reverse phase (C_18_) column (1·9 μm, 200 × 2·1 mm) or a Merck ZIC-pHILIC column (polymeric 5 μm, 150 × 4·6 mm) (see online Supplementary Material 4 for details on the chromatography column used for each dietary biomarker). Mass spectra were acquired using MRM acquisition, in positive and negative ionisation modes simultaneously. Collision energy and tube lens voltage values were individually optimised for each parent–product transition measured (see online Supplementary Material 4 for optimised values for each measured transition). All post-acquisition data processing was performed using Quan Browser (Thermo Scientific) and Xcalibur (Thermo Scientific).

### Analysis of non-targeted metabolite fingerprint data

Supervised classification of fingerprint data was performed using Random Forest (RF) classification using the randomForest package^(33)^ in R^(32)^. For all RF models, the number of trees (*ntree*) used was 1000, and the number of variables considered at each internal node (mtry) was the square root of the total number of variables. Accuracy, margins of classification and area under the receiver operator characteristic curve were all used to evaluate the performance of classification models, as described previously^(34)^. RF classification models were plotted following multidimensional scaling (MDS). Proximity measures for each individual observation were extracted from RF models and scaled coordinates produced using *cmdscale* on 1 – proximity.

### Analysis of quantitative data from targeted metabolite profiling

Kruskal–Wallis and paired *t* tests were used to determine significance differences between the classes. All *P*-values were corrected for multiple testing using Bonferroni correction.

## Results

In a preliminary experiment, we evaluated public perceptions of three different home collection methods for spot urine sampling, described in online Supplementary Material 1A–C. The results showed that all three procedures were perceived to be acceptable by the general public (results shown in online Supplementary Material 5), with no significant differences observed in the mean acceptability scores for each method (*P* = 0·85, Kruskal–Wallis test). In the current study, we evaluated vacuum tube technology utilising a separate transfer straw (see online Supplementary Material 1D), which offered scope to collect multiple spot urines in the home environment and post to an analytical facility, potentially without the need for refrigeration to preserve sample composition. The overall study comprised of three independent sub-studies (Fig. 1). *Sub-study 1* explored the need for chemical preservatives in vacuum tubes, while *sub-study 2* tested the performance of vacuum tube technology to maintain the compositional stability of urine samples in comparison to traditional methods, which required sample freezing; a range of storage treatments were evaluated, which mimicked conditions typically encountered in home environments. *Sub-study 3* used an online questionnaire in a free-living population to assess the acceptability of an optimised spot urine sampling method potentially suitable for large-scale epidemiological studies.

### Stability of urine metabolites after short- to medium-term storage in vacuum tubes maintained under different temperature regimes, with and without a preservative

**Fig. 2:**
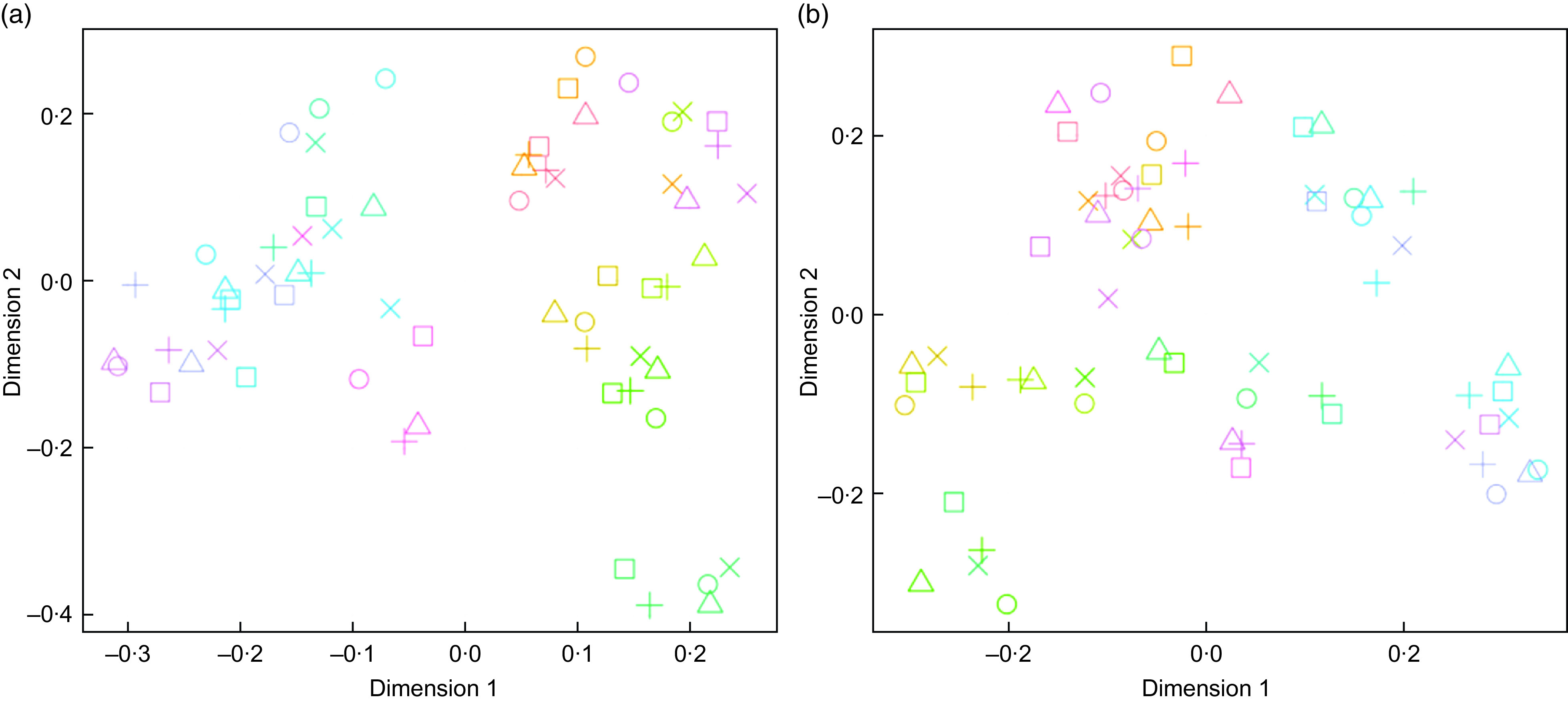
Multidimensional scaling plots of Random Forest proximity scores from supervised classification models of flow infusion electrospray ionisation high-resolution MS fingerprint data using storage treatment as the response value. Storage treatments were as follows: control, –20°C, T1, 2 d at 4°C, T2, 7 d at 4°C; T3, 2 d at room temperature (RT); T4, 7 d at RT. (a) Preservative-coated tubes, (b) non-preservative**-**coated tubes. Samples are coloured by individual, and shapes indicate treatment. Participant: 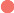, 1; 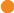, 2; 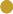, 3; 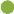, 4; 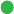, 5; 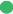, 6; 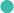, 7; 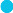, 8; 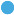, 9; 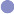, 10; 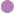, 11; 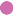, 12. Storage treatment: 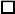, control; 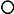, T1; 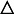, T2; 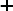, T3; 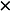, T4

FIE-HRMS fingerprints were generated for each urine sample, and multidimensional scaling plots of RF proximity scores from supervised classification models were used to determine whether the presence of a preservative had an impact on overall urine composition following exposure to storage regimes likely to be encountered during home collection spanning several days (Fig. 2). Most participants had distinctly individual urine metabolomes, with their urine samples after each different storage temperature regime clustering together (Fig. 2(a)). The inclusion of a preservative had little discernible impact on sample clustering patterns (Fig. 2(b)). An RF classification analysis of FIE-HRMS fingerprint data was used to quantify the overall compositional differences in binary comparisons between each treatment and the –20°C control (which mimicked storage in a typical domestic freezer). Classification accuracies and area under the receiver operator characteristic curve values <0·4 and RF margins <0·2 indicated that storage temperature regimes in either the coated or non-coated tube had no significant impact on the overall urine composition (Table 1).

**Table 1:** Summary statistics for binary classification by Random Forest (RF) of first morning void spot urine samples stored under different conditions within preservative-coated and non-coated vacuum tubes

Pairwise	Preservative-coated tube			Non-coated tube		
	Classification accuracy	AUC	RF classification margin	Classification accuracy	AUC	RF classification margin
Control *v*. 2 d at 4°C	0·30	0·24	–0·11	0·23	0·16	–0·16
Control *v*. 7 d at 4°C	0·26	0·20	–0·13	0·20	0·15	–0·18
Control *v*. 2 d at RT	0·30	0·26	–0·12	0·21	0·16	–0·16
Control *v*. 7 d at RT	0·31	0·25	–0·10	0·27	0·20	–0·14
2 d at 4°C *v*. 7 d at 4°C	0·29	0·25	–0·11	0·20	0·13	–0·19
2 d at 4°C *v*. 2 d at RT	0·31	0·28	–0·11	0·24	0·16	–0·16
2 d at 4°C *v*. 7 d at RT	0·30	0·24	–0·12	0·27	0·20	–0·14
7 d at RT *v*. 2 d at RT	0·27	0·19	–0·15	0·25	0·18	–0·15
7 d at 4°C *v*. 7 d at RT	0·29	0·23	–0·12	0·25	0·19	–0·14
2 d at RT *v*. 7 d at RT	0·28	0·23	–0·12	0·22	0·16	–0·16

Control, storage at –20°C; RT, room temperature; AUC, area under the receiver operating characteristic curve.

**Fig. 3:**
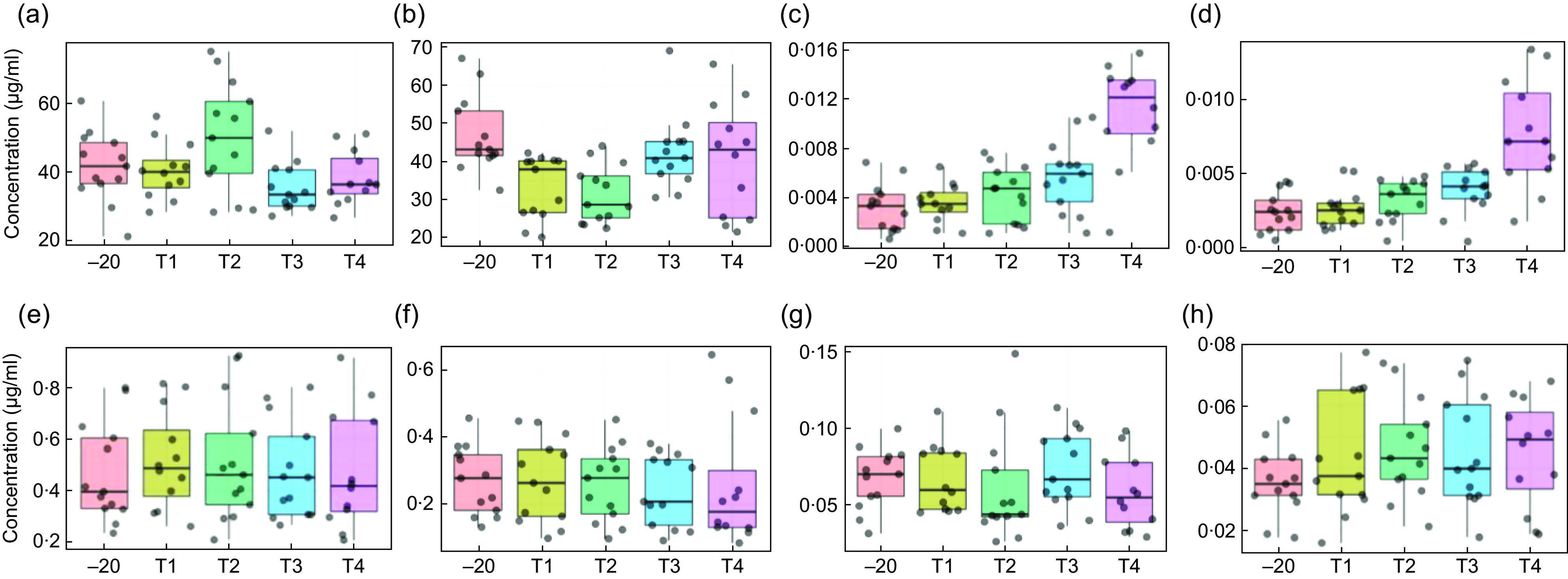
Box plots of selected dietary biomarkers showing stability in vacuum tubes and impact of preservatives after exposure to various storage conditions. VT, non-preservative-coated vacuum tube; CVT, preservative-coated vacuum tube. (a) VT–1-methyl-histidine; (b) CVT–1-methyl-histidine; (c) VT–daidzein; (d) CVT–daidzein; (e) VT–ferulic acid; (f) CVT–ferulic acid; (g) VT–tryptophan; (h) CVT–tryptophan. –20 (storage at –20°C); T1, 2 d at 4°C; T2, 7 d at 4°C; T3, 2 d at room temperature (RT); T4, 7 d at RT

The absolute concentrations of selected biomarkers **c**overing a wide range of foods were measured in urine samples collected in the presence or absence of chemical preservatives after exposure to a range of temperature regimes (see online Supplementary Material 6). In line with the previous results from metabolome fingerprinting, Fig. 3 shows, for selected biomarkers, that the main source of variance was individual participant, with only a small influence of storage regime. Statistical analysis (Kruskal–Wallis) of this data comparing the effect of all storage treatments on biomarker concentrations, in the presence or absence of a preservative (see online Supplementary Material 7), revealed that only four of the forty-six biomarkers (1-methyl-histidine, daidzein, ferulic acid and tryptophan) had a *P*-value <0·05 after correcting for multiple testing. In vacuum tubes with a lyophilised preservative, degradation of 1-methyl-histidine was evidenced after all storage conditions compared with the –20°C control. Daidzein concentration specifically was affected by storage at room temperature for 7 d (Fig. 3), showing an increase in concentration in both the uncoated vacuum tube and the vacuum tube with a lyophilised preservative. Ferulic acid increased in the uncoated vacuum tube after 2 d at 4°C. Tryptophan concentration significantly increased in the coated tubes after storage. In general, the presence of preservative had little additional impact on biomarker concentrations. Small but non-significant increases in the optical density of urine samples were evident after 2 d of incubation at room temperature (see online Supplementary Material 8) with little difference in microbial growth in vacuum tubes containing a preservative compared with non-coated vacuum tubes.

### Compositional analysis of spot urines collected and stored in a community setting using the traditional jug and universal tube method and the vacuum transfer system

**Fig. 4:**
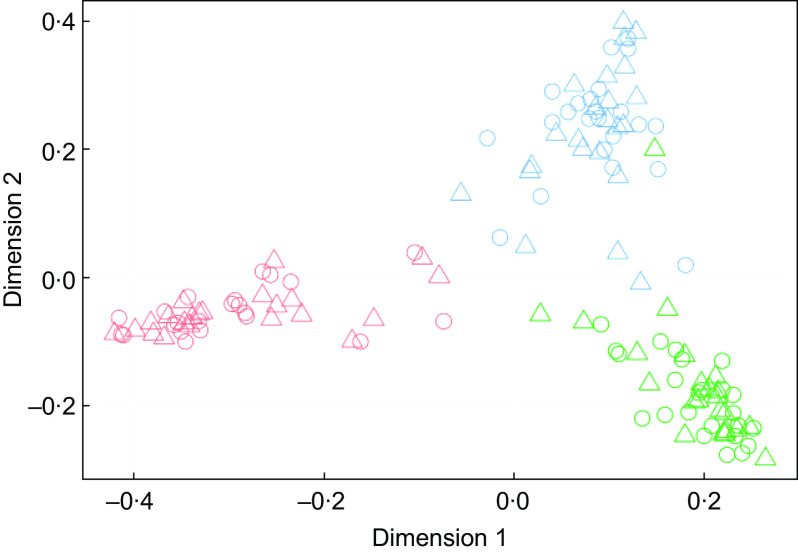
Multidimensional scaling plots of Random Forest proximity values from supervised classification models of flow infusion electrospray ionisation high-resolution MS of two different first morning void urine samples (universal tube and vacuum tube) over three different dietary intervention days. Collection method: 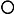, universal (jug); 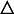, vacuum tube. Menu day: 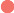, day 1; 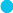, day 2; 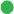, day 3

The chemical composition of urine samples collected in the home on three separate days using either a traditional jug and universal tube or a commercial vacuum transfer system (*sub-study 2*) were compared using metabolite fingerprinting. The different diets consumed on each of the three experimental days (see online Supplementary Material 2) were clearly evident in the FIE-HRMS data following MDS of RF proximity values (Fig. 4). However, the chemical fingerprint data of urine samples collected and stored using the two sampling methods overlapped for each day, indicating compositional similarity. The summary statistics for RF binary classification^(34)^ of FMV spot urine samples collected on each food intervention day (Table 2) indicate no detectable differences in the overall chemical composition of urine samples collected by the two different methods.

**Table 2:** Summary statistics for pairwise comparisons between first morning void urine samples collected using the universal and vacuum transfer method by Random Forest (RF) on three different food intervention days

Menu day	Classification accuracy	95 % CI	AUC	95 % CI	RF classification margin	95 % CI
Day 1	0·47	0·45, 0·50	0·47	0·44, 0·51	–0·02	–0·03, –0·01
Day 2	0·38	0·35, 0·40	0·36	0·33, 0·39	–0·06	–0·07, –0·05
Day 3	0·48	0·46, 0·50	0·48	0·46, 0·50	–0·02	–0·03, –0·01

AUC, area under the receiver operating characteristic curve.

The stability of exemplar dietary exposure biomarkers was examined in urine samples derived from both collection methods after absolute quantification using a targeted analysis method. These previously published dietary exposure biomarkers (see online Supplementary Material 4) represent a range of chemical classes for which standards are commercially available. The paired *t* test statistics (Table 3) revealed very little differences in biomarker concentrations (only seven biomarkers had an adjusted *P-*value ≤0·05; 1-methyl-histidine, 4-hydroxyhippuric-acid, hippuric-acid, proline-betaine, carnitine, tryptophan and ferulic acid-4-*O-*sulfate) between urine samples collected and stored using the two collection methods.

**Table 3:** Paired *t* tests to determine significant differences in biomarker concentrations between standard universal collection and vacuum transfer method, irrespective of menu or individual effects of fifteen participants who collected first morning void urine on three separate days in a home setting

Biomarker	*t* Statistic	*P* *
1-Methyl-histidine	–4·725	<0·001
3-Hydroxyhippuric-acid	2·644	0·506
3-Methyl-histidine	–2·465	0·828
3-Methyl-xanthine	–0·659	1·000
4-Hydroxyproline-betaine	–3·414	0·064
4-Hydroxyhippuric-acid	4·075	0·009
7-Methyl-xanthine	1·703	1·000
Acesulfame-K	–0·128	1·000
Anserine	0·536	1·000
BOA (1,3-benzoxazol-2-one)	–0·842	1·000
Caffeine	–2·564	0·636
Carnitine	–4·309	0·004
Carnosine	–0·664	1·000
Creatinine	–1·690	1·000
d-l-sulforaphane-glutathione	–0·555	1·000
d-l-sulforaphane-l-cysteine	1·632	1·000
d-l-sulforaphane-*N*-acetyl-l-cysteine	1·412	1·000
Daidzein	0·761	1·000
DHBA (3,5-dihydroxybenzoic acid)	–0·506	1·000
DHBA-3-*O*-sulfate	2·360	1·000
DHPPA (3-(3,5-Dihydroxyphenyl)-1-propanoic acid)	–1·381	1·000
DHPPA-3-sulfate	0·056	1·000
Epicatechin	0·794	1·000
Ferulic-acid	1·912	1·000
Ferulic-acid-4-*O*-b-d-glucuronide	–1·140	1·000
Ferulic-acid-4-*O*-sulfate	3·611	0·036
Hippuric-acid	7·391	<0·001
Indoxyl-sulphate	1·187	1·000
l-Phenylalanine	–1·660	1·000
Tryptophan	–3·582	0·039
*N*-2-Furoyl-glycine	0·939	1·000
Naringenin	–1·583	1·000
*p*-Cresol-glucuronide	–2·677	0·478
*p*-Cresol-sulphate	1·622	1·000
Phenyl-acetyl-l-glutamine	0·933	1·000
Proline-betaine	–3·955	0·012
Protocatechuic-acid	–2·518	0·736
Quercetin	1·067	1·000
Quercetin-3-*O*-b-d-glucuronide	1·277	1·000
Resveratrol	0·490	1·000
Rhamnitol	0·725	1·000
Sucrose	0·647	1·000
Tartarate	–1·574	1·000
Taurine	–1·561	1·000
Trigonelline	–2·414	0·920
Trimethylamine-*N*-oxide	–1·678	1·000

* All *P*-values are adjusted for multiple testing using Bonferroni correction.

### Evaluation of study participants’ acceptability of a postal method to collect urine samples in a community setting

**Fig. 5:**
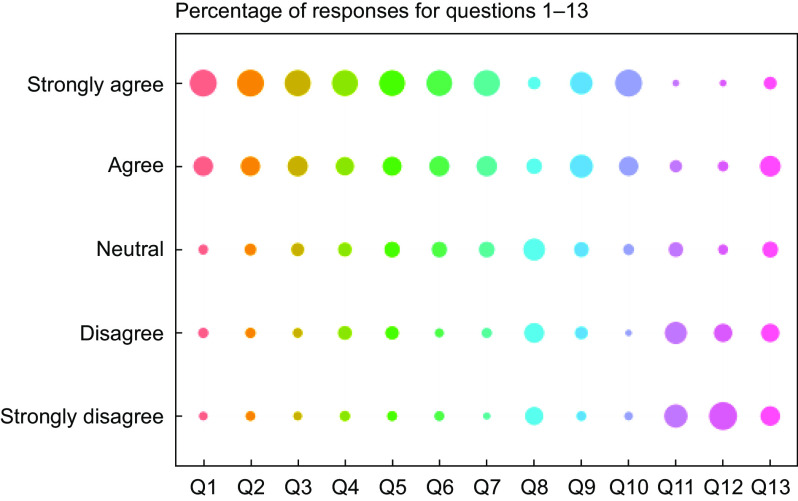
Summary (as a percentage of overall feedback) of responses to the thirteen self-recorded urine collection acceptability questions (see online Supplementary Material 5 for details). Responses (%): 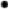, 0; 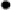, 20; 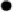, 40; 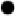, 60

The demonstration that overall urine composition was stable when stored for up to a week at 4°C in non-coated vacuum tubes and that the majority of dietary exposure biomarker concentrations were largely unaffected under these storage conditions offered the opportunity to explore the possibility of collecting urine samples in a community setting without the need to visit a CRC for sample drop-off. Free-living volunteers (*n* 122) were asked to follow their usual diet and to collect three FMV urine samples on random days over a week using the vacuum tube and transfer straw method (see online Supplementary Material 1D). Volunteers were asked to complete an online questionnaire assessing the acceptability of various steps in the process of collecting, storing and posting urine samples (see online Supplementary Material 3). Overall, the volunteers indicated a high acceptability of this method of home collection, storage and posting of urine samples (Fig. 5). The only question that showed a high ‘neutral’ response was ‘I would have preferred to collect urine samples at a different time of day’. In response to the last question (Q13), ‘I think collecting a urine sample out of the home environment is embarrassing’, 38 % of volunteers reported a negative answer (either agreeing or strongly agreeing with the statement) compared with only 2 % providing a negative response for Q12, ‘I think collecting urine samples in a home environment is embarrassing’. Although Q13 recorded the largest negative response, 49 % of recorded responses were positive with a further 13 % neutral.

## Discussion

A key observation in the current study is that spot urine samples collected using a vacuum transfer method are generally compositionally stable for several days in a domestic fridge or at room temperature, even in the absence of a chemical preservative. From a practical perspective, the collection procedure was highly acceptable to study participants, and the small (6 ml) vacuum tubes could be posted via the domestic mail system in the UK, avoiding the need to visit a CRC.

Urine provides a rich source of objective information on dietary intake (and other chemical exposures)^(18,20,28,35)^ and is a relatively non-invasive sample that participants can collect in their home settings. For high compliance to study protocols, urine collection methods need to be acceptable to participants, particularly with regard to hygiene and any adverse impact on normal daily activities. In an earlier work, we utilised a spot urine sampling methodology by participants in home settings using the traditional plastic jug followed by decanting of the sample into a smaller vessel (a 30-ml universal tube) for transport to the laboratory^(35,36)^. However, urine spillage may occur during decanting, resulting in a potential contamination of the outside of the transport vessel, and potentially exposing research staff and study participants/patients to microbial infection. Bespoke kits that avoid contamination during decanting are commercially available, including devices with a collection tube integrated into a ‘flow-through’ collection vessel (e.g., Peezy) and several alternative kits (e.g., Vacutest (Kima) and Vacutainer devices (Becton, Dickinson and Company) utilising a vacuum transfer system to draw up small volumes under partial vacuum via a needle into transport tubes (see online Supplementary Material 1). Using an online questionnaire, we demonstrated that members of the general public reported high and similar acceptability for all three methods proposed for spot urine collection (see online Supplementary Material 5). The vacuum transfer system was then explored in more detail as it offered, additionally, an opportunity to evaluate whether storage under vacuum would help preserve urine composition during storage in the home and transport without a need for maintaining a ‘cold chain’, thus greatly increasing logistical flexibility as well as reducing costs.

In *sub-study 1* we determined whether the use of vacuum tubes containing a lyophilised preservative would further improve stability when urine samples were exposed to range of conditions likely to be experienced during the collection and transport process. It is worth noting that the manufacturer suggests that the preservative stabilises urine over 72 h without the need for refrigeration; however, we tested storage at –20°C, refrigeration (2 and 7 d) and RT (2 and 7 d)^(37,38)^. Using QQQ MS MRM methodology, we discovered that the concentrations of a wide selection of dietary exposure biomarker signals after each storage condition were very similar, irrespective of the presence of a preservative. The data suggest that the majority of concentration changes occurring during storage were unlikely to be derived from bacterial activity. For example, the degradation of 1-methyl histidine in the vacuum tube with and without a lyophilised preservative was evident under all storage conditions compared with the –20°C control. Daidzein remained stable at 4°C with and without preservative, but its concentration increased after 2 and 7 d at RT, suggesting a possible breakdown of daidzein conjugates (glycines, glucuronides, sulfates and sulfoglucuronides) into aglycone^(39)^, again unrelated to bacterial activity. In contrast, ferulic acid, a major microbial degradation product of dietary polyphenols^(40)^, increased in the uncoated vacuum tube, which may reflect limited microbial activity. These observations support our hypothesis that the use of vacuum tube (without preservative) and straw collection method would minimise dietary biomarker degradation by avoiding ingress of contaminating microbes from the environment and – through a low level of oxygen in the tubes – limiting microbial growth and oxidative degradation of urine samples. The fact that the inclusion of a chemical preservative within the vacuum tubes provided little improvement in the stability of dietary intake biomarkers is an important consideration from an analytical perspective. This is because, due to the solubilisation of lyophilised coating containing chlorhexidine, ethyl paraben and sodium propionate, the concentrations of these compounds in the urine sample can vary depending on the volume of urine drawn into the tube. As a consequence, variable amounts of these compounds could dominate compositional differences between samples and might interfere with metabolome assessment. Other common urine preservatives that are strongly ionic, such as boric acid, might interfere with ionisation behaviour in MS and are to be avoided whenever possible (as reviewed by reference (41)).

Previous research has documented the impact of sample collection and storage conditions on the metabolic composition of human urine. In standard non-vacuum tubes, high-resolution metabolic fingerprinting has demonstrated that urinary metabolome is altered by storage at room temperature from 24 to 72 h^(26)^. In contrast, urine samples stored at –20°C exhibited global stability over a long period compared with urine stored at –80°C^(23,24)^. Other publications reported no major changes in urinary metabolite fingerprints when stored in non-vacuum tubes held at 4°C for up to 72 h^(26)^, but compositional modifications were observed with storage over longer periods^(23–25)^. Storage of urine in vacuum tubes at –85°C and then for up to 24 h at 4°C did not affect metabolic profiles assessed by NMR or GC-TOF-MS^(42–44)^. In *sub-study 2* we explored the effects of storage conditions on urine samples by simulating possible ‘real-life’ situations in which urine was stored 4°C for up to 4 d (simulating storage at home in a domestic fridge). Initial observations indicated that the impact of storage conditions on urinary metabolome was much smaller than the distinctive inter-individual differences in urine metabolome when we compared specifically the overall chemical composition of urine samples collected by the traditional jug and universal tube method (followed by immediate freezing at –80°C) with a commercial vacuum transfer system. The data indicated no major differences in overall chemical fingerprints of urine samples between methods on each of the three intervention days during which different foods were consumed. Additionally, there were no concentration differences for the majority of a large range of dietary exposure biomarkers covering multiple chemical classes. Overall, evidence shows that when using the vacuum transfer system, the patterns and concentrations of key dietary biomarkers and chemical groups were stable over several days in a domestic fridge without the need for immediate freezing.

It is unlikely that a single spot urine sample can provide robust data on habitual dietary intake at the individual level, and this issue is particularly acute for foods that are eaten infrequently and/or at irregular intervals. Additionally, as excretion kinetics may also differ between biomarkers^(19)^, there may be a need to take samples at more than one time-point in a study day to ensure measurement sensitivity. Therefore, to provide data on habitual exposure, multiple spot urine samples over several days would need to be collected and stored by participants at home before transport to the research facility. In *sub-study 3* we tested the acceptability of this idea by posting urine collection kits to free-living participants (*n* 122) who were asked to follow their usual diet and to collect three FMV urine samples at home (using the vacuum tube and transfer straw method) on random days over a week and then post samples back to the research centre. The acceptability of this protocol for urine collection, storage and posting of urine samples was high, and there was evidence that collecting multiple samples in a home environment was preferable over visiting a clinical research facility for sample collection.

To conclude, we developed and tested a spot urine collection methodology for the analysis of dietary exposure biomarkers. We demonstrated that this methodology is acceptable to the general public for use at home and in community settings. To assist with the evaluation of habitual dietary exposure, multiple spot urine samples can be collected at home throughout a typical week and stored in the fridge, without a risk of significant degradation of metabolite composition. In addition, the vacuum tubes containing urine samples can be posted directly, without the need for preservatives or refrigeration and without the involvement of clinical professionals, to an analytical facility for archiving and subsequent analyses.

## Supplementary material

For supplementary material accompanying this paper visit https://doi.org/10.1017/S136898002000097X. click here to view supplementary material
