# Supplementary material for: Developing community-based urine sampling methods to deploy biomarker technology for the assessment of dietary exposure
Source: Public Health Nutr. 2020 Jun 11;23(17):3081–92. doi: 10.1017/S136898002000097X (PMC10200380; doi:10.1017/S136898002000097X)
Supplement: Supplementary file 1 [file S136898002000097Xsup001.doc]

**Supplementary** **Material 1:** Assessing commonly used and novel methods of spot urine sample collection and storage

An internet search was carried out to identify commonly used and novel methods of spot urine sample collection and storage. Four methods were selected which could provide urine samples suitable for biomarker discovery/dietary exposure validation, based on their perceived suitability for use in the community and on the commercial availability of the collection devices. The first method (**A**) followed a standard practice of collecting urine in a 500 mL plastic jug followed by decanting a sample into a smaller vessel, in this case a 30 mL Universal tube, as described in previous studies . Two further methods utilised specific urine sampling tools including the ‘Peezy’ urine collection device (Peezy, Funnelly Enough Ltd, London, UK) which facilitated transfer of a sample into a 30 mL Universal tube (**B**), and a vacuum transfer system (**C**) using a 60 mL collection container with an integral transfer needle and a 6 mL additive-free vacuum tube (Vacutest, Kima). (**D**) A variant of (**C**) used a separate transfer straw to contact the urine sample.

Images of traditional jug and Universal tube, Peezy and vacuum transfer urine collection methods

(D)

(A) a standard 500 mL plastic jug and aliquoting into a 30 mL Universal tube; (B) the ‘Peezy’ urine collection device into a 30mL Universal tube; (C) vacuum transfer system using a 60 mL collection container with integral needle and a 6 mL vacuum tube. (D) Vacuum transfer system using a 100 mL collection container, a separate urine transfer straw and a 6 mL vacuum tube

**Supplementary** **Material 2** Meal plans in ***Sub-study (2)*** ^(29)^

| **Menu plan: Food intervention Day 1** |
| --- |
| **Breakfast:** Coffee, sourdough rye bread toasted, sweetened breakfast cereal and milk, banana |
| **Lunch:** Coffee, Tuna and sweetcorn salad on sourdough rye bread, banana |
| **Dinner:** Salmon, Broccoli and chips. Almonds and wine |
|  |
| **Menu plan: Food intervention Day 2** |
| **Breakfast:** Tea, wholegrain bread toasted, red berries, milk |
| **Lunch:** Tea, wholegrain bread, cheese, ham, carrot, berries, |
| **Dinner:** Spinach, mushrooms, potato, steak pie, lager, raisins , milk |
|  |
| **Menu plan: Food intervention Day 3** |
| **Breakfast:** White bread, porridge, milk, egg, bacon, apple juice, cocoa |
| **Lunch:** White bread, salami, pepper, cocoa, apple |
| **Dinner:** Chicken curry, rice, peas, wine, cocoa |

(29) Lloyd AJ, Willis ND, Wilson T *et al.* (2019) Developing a Food Exposure and Urine Sampling Strategy for Dietary Exposure Biomarker Validation in Free-Living Individuals. *Mol Nutr Food Res*. **63**(**14**), 1900062.

**Supplementary** **Material 3:** Vacuum transfer system questionnaire on acceptability of the postal method for sample return

The questions are asked in a way to rate your opinion towards a statement concerning the method on a scale from **strong agreement** to **strong disagreement** with the statement. Please estimate your opinion to the stated as best as you can and fill in the circle that is closest to match with it. Fill-in only one circle for each question.

Neither agree nor disagree

Strongly disagree

Disagree

Agree

Strongly agree

| **Questions** |
| --- |
|  |
| 1. I was successful in collecting urine using this method |
| 2. It was easy to collect urine in the pot |
| 3. I was confident collecting urine in the pot |
| 4. It was easy to transfer urine from the pot into the capped tube using the straw |
| 5. I felt confident transferring urine from the pot into the capped tube using the straw |
| 6. I would be happy to write the collection date and time on the capped tube |
| 7. I was happy collecting first morning void samples |
| 8. I would have preferred to collect urine samples at a different time of day |
| 9. I was happy storing several urine samples collected over a week in my fridge |
| 10. I was happy to post urine samples in a pre-paid box |
| 11. In general, I think collecting an urine sample is difficult |
| 12. I think collecting an urine sample in a home environment is embarrassing |
| 13. I think collecting an urine sample OUT of the home environment is embarrassing |
|  |

**Supplementary** **Material 4**: Biomarkers used for absolute quantification in urine samples

| **Biomarker** | **Dietary component** | **Column** | **Ionisation mode^a^** | **Parent ion^b^** | **Product ion^c^** | **Retention time** |
| --- | --- | --- | --- | --- | --- | --- |
| 1-Methyl-histidine | Striated muscle meat | pHILIC | Pos | 170.064 | 124.160 | 9.9 |
| 3-Hydroxyhippuric-acid | Fruit and Vegetables | RP-C18 | Neg | 194.064 | 150.113 | 5.12 |
| 3-Methyl-histidine | Poultry and fish (no shellfish) | pHILIC | Pos | 170.059 | 96.351 | 10.6 |
| 3-Methyl-xanthine | Cocoa (chocolate) | RP-C18 | Pos | 167.096 | 94.220 | 4.52 |
| 4-Hydroxyhippuric-acid | Fruit and Vegetables | RP-C18 | Neg | 194.073 | 100.240 | 4.84 |
| 4-Hydroxyproline-betaine | Citrus and Citrus fruit juice | pHILIC | Pos | 160.167 | 88.367 | 9.4 |
| 7-Methyl-xanthine | Cocoa (chocolate) | RP-C18 | Pos | 167.085 | 124.185 | 4.36 |
| Acesulfame-K | Low calorie drinks | RP-C18 | Neg | 161.947 | 82.233 | 4.32 |
| Anserine | Poultry and fish (no shellfish) | pHILIC | Pos | 241.052 | 109.159 | 11.1 |
| BOA (1-3-Benzoazol-2-one) | Wholegrain rye | RP-C18 | Pos | 136.055 | 80.292 | 6.86 |
| Caffeine | Cocoa (chocolate), Coffee, Tea, Caffeinated drinks | RP-C18 | Pos | 195.060 | 138.138 | 5.94 |
| Carnitine | Striated muscle meat | pHILIC | Pos | 162.153 | 103.301 | 9.9 |
| Carnosine | Striated muscle meat | pHILIC | Pos | 227.064 | 110.202 | 12 |
| Creatinine | Striated muscle meat | pHILIC | Pos | 114.129 | 44.695 | 8.5 |
| Daidzein | soy, legumes | RP-C18 | Pos | 255.057 | 199.183 | 7.52 |
| DHBA | Wholegrain | RP-C18 | Neg | 153.010 | 109.094 | 4.63 |
| DHBA-3-O-sulfate | Wholegrain | RP-C18 | Neg | 232.880 | 152.988 | 3.99 |
| DHPPA | Wholegrain | RP-C18 | Neg | 181.044 | 137.132 | 5.37 |
| DHPPA-3-sulfate | Wholegrain | pHILIC | Neg | 260.950 | 137.124 | 16.3 |
| D-L-Sulforaphane-glutathione | Cruciferous vegetable e.g. Broccoli | RP-C18 | Pos | 485.049 | 355.919 | 5.18 |
| D-L-Sulforaphane-L-cysteine | Cruciferous vegetable e.g. Broccoli | RP-C18 | Pos | 299.003 | 114.103 | 4.77 |
| D-L-Sulforaphane-N-acetyl-L-cysteine | Cruciferous vegetable e.g. Broccoli | RP-C18 | Pos | 341.014 | 114.098 | 5.82 |
| Epicatechin | Berries, Drupes, Apple, Cocoa, Coffee, Green/black tea, Vegetables e.g. beans. | RP-C18 | Pos | 291.084 | 139.070 | 5.74 |
| Ferulic-acid | Coffee, polyphenol-rich foods | RP-C18 | Pos | 195.051 | 145.047 | 6.83 |
| Ferulic-acid-4-O-b-D-glucuronide | Coffee, polyphenol-rich foods | RP-C18 | Neg | 369.054 | 193.042 | 5.44 |
| Ferulic-acid-4-O-sulfate | Coffee, polyphenol-rich foods | RP-C18 | Neg | 273.021 | 193.087 | 5.59 |
| Hippuric-acid | Fruit and Vegetables | RP-C18 | Pos | 180.075 | 105.180 | 5.92 |
| Indoxyl-sulfate | Protein intake | RP-C18 | Neg | 212.012 | 132.155 | 5.28 |
| Phenylalanine | Protein intake | pHILIC | Pos | 166.131 | 120.279 | 8.9 |
| Tryptophan | Protein intake | pHILIC | Pos | 205.124 | 188.157 | 9.7 |
| N-2-Furoyl-glycine | Strongly heated foods | RP-C18 | Pos | 170.092 | 95.296 | 4.9 |
| Naringenin | Grapefruit, Citrus (orange, lemon, lime) | RP-C18 | Neg | 271.083 | 151.076 | 7.78 |
| p-Cresol-glucuronide | Protein intake | pHILIC | Neg | 283.033 | 107.163 | 8.1 |
| p-Cresol-sulfate | Protein intake | RP-C18 | Neg | 187.026 | 107.228 | 6.31 |
| Phenyl-acetyl-L-glutamine | Protein intake | pHILIC | Pos | 265.105 | 130.233 | 6 |
| Proline-betaine | Citrus and Citrus fruit juice | pHILIC | Pos | 144.190 | 58.543 | 8.3 |
| Protocatechuic-acid | Red wine and other plants sources | RP-C18 | Neg | 153.080 | 109.193 | 4.86 |
| Quercetin | Fruits (e.g., apples, grapes, berries), Vegetables (e.g., onions, spinach, kale, broccoli, lettuce, and tomatoes), tea | RP-C18 | Neg | 301.002 | 151.032 | 7.58 |
| Quercetin-3-O-b-D-glucuronide | Fruits (e.g., apples, grapes, berries) vegetables (e.g., onions, spinach, kale, broccoli, lettuce, and tomatoes), tea | RP-C18 | Neg | 477.076 | 300.972 | 6.66 |
| Resveratrol | Red wine, grapes | RP-C18 | Pos | 229.089 | 107.199 | 7.49 |
| Rhamnitol | Apple | pHILIC | Neg | 165.146 | 59.139 | 8.6 |
| Sucrose | High sugar intake | pHILIC | Neg | 341.101 | 89.039 | 10.9 |
| Tartarate | Grapes and wine | pHILIC | Neg | 149.016 | 87.191 | 13.9 |
| Taurine | Striated muscle meat | pHILIC | Pos | 126.126 | 108.255 | 11.8 |
| Trigonelline | Legumes: Beans, soya, peanuts, almonds, coffee, peas | pHILIC | Pos | 138.023 | 92.259 | 8.9 |
| Trimethylamine-N-oxide | Fish | pHILIC | Pos | 76.188 | 58.517 | 9.7 |
| 4-Chloro-DL-phenylalanine | IS | pHILIC | Pos | 200.042 | 154.112 | 8.5 |
| Syringic acid | IS | RP-C18 | Pos | 199.060 | 140.047 | 6.12 |

***a:*** ions denoted with (pos) or (neg) indicates that the biomarker was detected in the protonated or deprotonated form respectively.

***b:*** All parent ions were detected as either the protonated (M+H) or deprotonated (M-H) form of the mono-isotopic mass (M) of each biomarker.

***c:*** For each parent ion; a minimum of three product ions were detected and analysed. The product ions shown are the ones which demonstrated the greatest stability and were therefore used for quantification. The remaining product ions (not shown) were used as qualifying ions only.

**Supplementary** **Material 5**: Perception of different spot urine collection methods

*Methods*

In total, 31 individuals (20 female, 11 male; age: 20-73) were recruited from a database maintained at the Human Nutrition Research Centre, Newcastle University of individuals who had taken part previously, or had expressed an interest, in nutrition studies. These participants were asked to complete an online questionnaire consisting of six questions (below) about their perceptions of the three spot urine collection methods (**Supplementary** **Material 1, A-C**) which required them to rank the extent of their agreement with each statement on a five point scale from “strongly disagree” to “strongly agree”. The Kruskal-Wallis Test was used to compare sample collection methods and to calculate *p*-values.

*Online questionnaire*

The questions are asked in a way to rate your opinion towards a statement concerning the method on a scale from **strong agreement** to **strong disagreement** with the statement. Please estimate your opinion to the stated as best as you can and fill in the circle that is closest to match with it. Fill-in only one circle for each question.


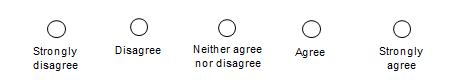


**Question 1:** I understand how the collection of urine with Method x works

**Question 2:** I think it would be easy to collect urine with Method x

**Question 3:** I would feel confident collecting urine with Method x

**Question 4:** I think that the urine transfer step of Method x is easy

**Question 5:** I would feel comfortable performing the urine transfer with Method x

**Question 6:** I would be happy to label the tubes used in Method x

*Results of Questionnaire*

The results showed that all three procedures were perceived to be acceptable by the general public with a mean score greater than 4, where the maximum was 5. Although no significant differences were observed in the mean acceptability scores for each method (*p*-value 0.85, Kruskal- Wallis Test), there was some variance in the participant perception of the methods, depending on factors such as age and gender (data not shown).

**Supplementary** **Material 6**: Absolute quantification in selected first morning void (FMV) urine samples stored in coated and non-coated vacuum tubes.

|  | Coated vacuum tubes | | | | | Non-Coated vacuum tubes | | | | |
| --- | --- | --- | --- | --- | --- | --- | --- | --- | --- | --- |
| Biomarker | Control | T1 | T2 | T3 | T4 | Control | T1 | T2 | T3 | T4 |
| 1-Methyl-histidine | 46.94 | 33.30 | 31.43 | 42.37 | 40.48 | 41.61 | 40.44 | 49.91 | 35.41 | 38.28 |
| 3-Hydroxyhippuric-acid | 7.67 | 7.49 | 6.27 | 5.59 | 6.29 | 8.78 | 9.55 | 9.29 | 8.30 | 7.28 |
| 3-Methyl-histidine | 62.17 | 53.80 | 51.69 | 59.59 | 55.63 | 65.45 | 46.09 | 66.73 | 53.51 | 36.97 |
| 3-Methyl-xanthine | 2.61 | 2.63 | 2.42 | 2.47 | 2.67 | 3.18 | 3.15 | 3.27 | 3.13 | 3.58 |
| 4-Hydroxyproline-betaine | 8.34 | 6.11 | 5.46 | 7.93 | 7.38 | 7.89 | 8.97 | 7.16 | 6.73 | 9.61 |
| 4-Hydroyhippuric-acid | 9.33 | 9.91 | 9.15 | 8.69 | 7.89 | 9.99 | 10.80 | 10.57 | 10.04 | 11.67 |
| 7-Methyl-xanthine | 7.38 | 7.49 | 7.58 | 7.17 | 7.37 | 8.32 | 8.13 | 8.39 | 8.14 | 8.96 |
| Acesulfame-K | 10.77 | 10.55 | 10.67 | 10.10 | 11.29 | 13.86 | 12.59 | 14.46 | 13.63 | 12.56 |
| Anserine | 3.97 | 3.82 | 3.82 | 4.31 | 3.75 | 4.52 | 3.46 | 4.06 | 3.82 | 2.77 |
| BOA-1-3-Benzoazol-2-one | 0.02 | 0.02 | 0.01 | 0.01 | 0.01 | 0.01 | 0.01 | 0.01 | 0.01 | 0.01 |
| Caffeine | 0.35 | 0.37 | 0.35 | 0.33 | 0.34 | 0.38 | 0.38 | 0.38 | 0.38 | 0.47 |
| Carnitine | 19.50 | 15.45 | 14.21 | 17.84 | 21.33 | 21.29 | 20.01 | 23.03 | 16.00 | 22.13 |
| Carnosine | 1.08 | 1.11 | 1.18 | 1.25 | 1.33 | 1.35 | 1.40 | 1.32 | 1.36 | 1.45 |
| Creatinine | 2651.11 | 2768.50 | 2861.50 | 3266.37 | 2903.09 | 3589.09 | 3506.62 | 3516.80 | 3131.94 | 3397.63 |
| D-L-Sulforaphane-glutathione | 0.01 | 0.01 | 0.01 | 0.01 | 0.01 | 0.01 | 0.01 | 0.01 | 0.00 | 0.01 |
| D-L-Sulforaphane-L-cysteine | 0.02 | 0.02 | 0.02 | 0.01 | 0.01 | 0.02 | 0.01 | 0.02 | 0.01 | 0.01 |
| D-L-Sulforaphane-N-acetyl-L-cysteine | 0.34 | 0.36 | 0.33 | 0.33 | 0.25 | 0.37 | 0.22 | 0.38 | 0.34 | 0.23 |
| Daidzein | <0.01 | <0.01 | <0.01 | <0.01 | 0.01 | <0.01 | <0.01 | <0.01 | 0.01 | 0.01 |
| DHBA | 0.47 | 0.49 | 0.48 | 0.52 | 0.48 | 0.50 | 0.51 | 0.53 | 0.53 | 0.55 |
| DHBA-3-O-sulfate | 1.98 | 2.02 | 1.83 | 1.77 | 1.74 | 2.76 | 2.85 | 2.80 | 2.76 | 3.12 |
| DHPPA | 0.25 | 0.27 | 0.26 | 0.29 | 0.27 | 0.30 | 0.30 | 0.30 | 0.28 | 0.30 |
| DHPPA-3-sulfate | 0.50 | 0.51 | 0.52 | 0.50 | 0.47 | 0.52 | 0.46 | 0.52 | 0.51 | 0.49 |
| Epicatechin | 0.02 | 0.02 | 0.03 | 0.02 | 0.02 | 0.02 | 0.03 | 0.02 | 0.02 | 0.02 |
| Ferulic-acid | 0.02 | 0.02 | 0.01 | 0.01 | 0.02 | 0.01 | 0.01 | 0.01 | 0.01 | 0.02 |
| Ferulic-acid-4-O-b-D-glucuronide | 0.27 | 0.27 | 0.26 | 0.24 | 0.26 | 0.47 | 0.52 | 0.51 | 0.48 | 0.48 |
| Ferulic-acid-4-O-sulfate | 0.96 | 1.02 | 1.01 | 0.93 | 1.01 | 1.31 | 1.33 | 1.40 | 1.37 | 1.49 |
| Hippuric-acid | 336.77 | 346.59 | 340.10 | 337.65 | 354.02 | 455.81 | 488.98 | 502.83 | 487.07 | 376.78 |
| Indoxyl-sulfate | 14.80 | 15.68 | 15.95 | 15.28 | 14.46 | 14.71 | 15.29 | 15.00 | 15.07 | 16.94 |
| Phenylalanine | 4.01 | 4.04 | 4.21 | 4.96 | 4.95 | 5.51 | 5.45 | 5.26 | 4.78 | 5.22 |
| Tryptophan | 7.36 | 5.41 | 4.85 | 6.97 | 6.89 | 6.69 | 7.71 | 7.98 | 6.22 | 7.01 |
| N-2-Furoyl-glycine | 3.19 | 3.34 | 3.52 | 3.15 | 0.91 | 4.07 | 4.06 | 4.24 | 3.87 | 4.11 |
| Naringenin | <0.01 | <0.01 | <0.01 | <0.01 | 0.01 | <0.01 | <0.01 | <0.01 | <0.01 | 0.01 |
| p-Cresol-glucuronide | 7.13 | 6.40 | 6.32 | 7.45 | 8.21 | 7.98 | 7.61 | 8.03 | 6.29 | 7.57 |
| p-Cresol-sulfate | 26.59 | 28.58 | 28.57 | 27.60 | 27.47 | 29.65 | 31.20 | 31.28 | 30.32 | 37.30 |
| Phenyl-acetyl-L-glutamine | 51.26 | 54.74 | 56.14 | 65.58 | 79.80 | 90.75 | 70.83 | 63.75 | 68.82 | 93.59 |
| Proline-betaine | 34.76 | 32.85 | 32.10 | 37.03 | 35.14 | 39.69 | 41.48 | 37.49 | 36.59 | 47.95 |
| Protocatechuic-acid | 0.12 | 0.13 | 0.13 | 0.14 | 0.12 | 0.13 | 0.13 | 0.13 | 0.14 | 0.13 |
| Quercetin | <0.01 | <0.01 | <0.01 | 0.01 | <0.01 | <0.01 | 0.01 | <0.01 | <0.01 | <0.01 |
| Quercetin-3-O-b-D-glucuronide | 0.12 | 0.14 | 0.14 | 0.27 | 0.11 | 0.11 | 0.12 | 0.11 | 0.13 | 0.15 |
| Resveratrol | 0.04 | 0.04 | 0.05 | 0.05 | 0.04 | 0.07 | 0.07 | 0.06 | 0.07 | 0.06 |
| Rhamnitol | 1.93 | 2.14 | 2.12 | 2.44 | 2.16 | 2.63 | 3.19 | 2.62 | 2.64 | 3.17 |
| Sucrose | 4.92 | 4.89 | 4.57 | 5.33 | 5.06 | 4.89 | 5.40 | 5.37 | 4.80 | 3.48 |
| Tartarate | 36.29 | 30.95 | 29.16 | 42.50 | 38.77 | 49.03 | 48.49 | 45.41 | 40.89 | 40.24 |
| Taurine | 5.67 | 5.74 | 6.61 | 6.32 | 6.39 | 8.12 | 8.40 | 7.41 | 7.20 | 6.66 |
| Trigonelline | 36.74 | 42.87 | 43.06 | 48.86 | 48.12 | 54.45 | 48.17 | 52.13 | 47.86 | 53.29 |
| Trimethylamine-N-oxide | 18.44 | 17.01 | 16.60 | 27.68 | 23.62 | 30.58 | 29.68 | 39.96 | 34.53 | 28.74 |

Values are means (µg / mL) from 13 individuals.

Where: Control, -20 °C; T1, 2 days at 4 °C; T2, 7 days at 4 °C; T3, 2 days at room temperature (RT); T4, 7 days at RT.

**Supplementary** **Material 7**: **S**tatistical analysis (Kruskal-Wallis) of absolute concentrations of dietary exposure biomarkers in selected first morning void (FMV) urine samples stored in coated vacuum tubes and vacuum tubes containing a lyophilized preservative*

| Biomarker | Coated vacuum tube | | | Non-coated vacuum tube | | |
| --- | --- | --- | --- | --- | --- | --- |
|  | Statistic | *P*-value | *p*-value (corrected) | Statistic | *p*-value | *p*-value (corrected) |
| 1-Methyl-histidine | 18.03 | **0.001** | 0.056 | 8.02 | 0.091 | 1.000 |
| 3-Hydroxyhippuric-acid | 0.58 | 0.965 | 1.000 | 0.41 | 0.982 | 1.000 |
| 3-Methyl-histidine | 1.82 | 0.769 | 1.000 | 1.85 | 0.763 | 1.000 |
| 3-Methyl-xanthine | 0.04 | 1.000 | 1.000 | 0.36 | 0.985 | 1.000 |
| 4-Hydroxyproline-betaine | 0.26 | 0.992 | 1.000 | 0.10 | 0.999 | 1.000 |
| 4-Hydroyhippuric-acid | 1.74 | 0.784 | 1.000 | 0.36 | 0.986 | 1.000 |
| 7-Methyl-xanthine | 0.12 | 0.998 | 1.000 | 0.30 | 0.990 | 1.000 |
| Acesulfame-K | 0.12 | 0.998 | 1.000 | 0.25 | 0.993 | 1.000 |
| Anserine | 0.46 | 0.977 | 1.000 | 0.98 | 0.913 | 1.000 |
| BOA-1-3-Benzoazol-2-one | 9.13 | 0.058 | 1.000 | 1.66 | 0.797 | 1.000 |
| Caffeine | 0.17 | 0.997 | 1.000 | 0.39 | 0.983 | 1.000 |
| Carnitine | 2.25 | 0.689 | 1.000 | 0.96 | 0.917 | 1.000 |
| Carnosine | 1.55 | 0.817 | 1.000 | 0.41 | 0.981 | 1.000 |
| Creatinine | 1.98 | 0.739 | 1.000 | 1.11 | 0.893 | 1.000 |
| D-L-Sulforaphane-glutathione | 0.67 | 0.955 | 1.000 | 4.68 | 0.322 | 1.000 |
| D-L-Sulforaphane-L-cysteine | 3.99 | 0.407 | 1.000 | 2.95 | 0.565 | 1.000 |
| D-L-Sulforaphane-N-acetyl-L-cysteine | 0.64 | 0.959 | 1.000 | 0.45 | 0.978 | 1.000 |
| Daidzein | 22.62 | **0.000** | **0.007** | 23.26 | **0.000** | **0.005** |
| DHBA | 0.44 | 0.979 | 1.000 | 0.32 | 0.989 | 1.000 |
| DHBA-3-O-sulfate | 0.75 | 0.945 | 1.000 | 0.67 | 0.955 | 1.000 |
| DHPPA | 0.44 | 0.979 | 1.000 | 0.40 | 0.982 | 1.000 |
| DHPPA-3-sulfate | 0.52 | 0.972 | 1.000 | 0.16 | 0.997 | 1.000 |
| Epicatechin | 3.32 | 0.506 | 1.000 | 1.87 | 0.760 | 1.000 |
| Ferulic-acid | 4.05 | 0.399 | 1.000 | 17.59 | **0.001** | 0.068 |
| Ferulic-acid-4-O-b-D-glucuronide | 1.43 | 0.840 | 1.000 | 0.92 | 0.922 | 1.000 |
| Ferulic-acid-4-O-sulfate | 0.28 | 0.991 | 1.000 | 0.49 | 0.975 | 1.000 |
| Hippuric-acid | 0.31 | 0.989 | 1.000 | 3.34 | 0.502 | 1.000 |
| Indoxyl-sulfate | 0.39 | 0.983 | 1.000 | 0.30 | 0.990 | 1.000 |
| Phenylalanine | 7.00 | 0.136 | 1.000 | 2.02 | 0.731 | 1.00 |
| Tryptophan | 11.93 | **0.018** | 0.821 | 4.22 | 0.377 | 1.000 |
| N-2-Furoyl-glycine | 2.59 | 0.629 | 1.000 | 0.30 | 0.990 | 1.000 |
| Naringenin | 8.50 | 0.075 | 1.000 | 7.06 | 0.133 | 1.000 |
| p-Cresol-glucuronide | 1.62 | 0.806 | 1.000 | 0.82 | 0.935 | 1.000 |
| p-Cresol-sulfate | 0.26 | 0.992 | 1.000 | 0.70 | 0.952 | 1.000 |
| Phenyl-acetyl-L-glutamine | 6.50 | 0.165 | 1.000 | 5.53 | 0.237 | 1.000 |
| Proline-betaine | 0.19 | 0.996 | 1.000 | 0.19 | 0.996 | 1.000 |
| Protocatechuic-acid | 1.39 | 0.847 | 1.000 | 1.14 | 0.887 | 1.000 |
| Quercetin | 0.96 | 0.916 | 1.000 | 0.92 | 0.921 | 1.000 |
| Quercetin-3-O-b-D-glucuronide | 0.37 | 0.985 | 1.000 | 0.20 | 0.995 | 1.000 |
| Resveratrol | 3.35 | 0.502 | 1.000 | 5.16 | 0.271 | 1.000 |
| Rhamnitol | 0.78 | 0.941 | 1.000 | 1.24 | 0.871 | 1.000 |
| Sucrose | 1.00 | 0.910 | 1.000 | 4.26 | 0.372 | 1.000 |
| Tartarate | 0.46 | 0.977 | 1.000 | 0.23 | 0.994 | 1.000 |
| Taurine | 0.53 | 0.970 | 1.000 | 0.71 | 0.950 | 1.000 |
| Trigonelline | 1.08 | 0.898 | 1.000 | 0.34 | 0.987 | 1.000 |
| Trimethylamine-N-oxide | 5.62 | 0.229 | 1.000 | 4.59 | 0.332 | 1.000 |

- Becton and Dickinson Vacutainer® urinalysis preservative tube; chlorhexidine, ethyl paraben and sodium propionate.

All storage treatments: Control, -20 °C; T1, 2 days at 4 °C; T2, 7 days at 4 °C; T3, 2 days at room temperature (RT); T4, 7 days at RT as in **Supplementary Material 6**. Significant *p-values* highlighted in bold and yellow

**Supplementary** **Material 8:** Box-plots of Optical Density (OD) (at 600 nm) of urine samples after different storage treatments

Where: T1, 2 days at 4 °C; T2, 7 days at 4 °C; T3, 2 days at room temperature (RT); T4, 7 days at RT.
